# Hydrophobic Ion Pairing with Cationic Surfactant Modifies Surface Activity and Colloidal Behavior of PFAS

**DOI:** 10.1021/acsestwater.6c00707

**Published:** 2026-08-29

**Authors:** Bishnu Pandey, Ligaya Manalastas, Afaq Ahmad, Siwei Gu, William H. Pennock, Arjun K. Venkatesan

**Affiliations:** Department of Civil & Environmental Engineering, 5965 New Jersey Institute of Technology, 323 Dr. Martin Luther King Jr. Blvd., Newark, New Jersey 07102, United States

**Keywords:** per- and polyfluoroalkyl substances, quaternary ammonium compounds, hydrophobic ion pairing, premicellar aggregation, surface tension, supramolecular assemblies, foam fractionation

## Abstract

Quaternary ammonium compounds (QACs) are increasingly used to enhance the removal of per- and polyfluoroalkyl substances (PFAS), particularly short-chain compounds, through hydrophobic ion pairing (HIP). Here, we investigated how HIP with cetyltrimethylammonium chloride (CTAC) influences the interfacial and colloidal behavior of selected PFAS at low-micromolar concentrations below reported critical micelle concentrations (CMCs) and across varying solution chemistries (pH, ionic strength, and natural organic matter) using surface tension measurements, dynamic light scattering (DLS), ζ-potential analysis, and transmission electron microscopy (TEM). Relative to the corresponding PFAS-only controls, CTAC (6.66 μM) reduced the surface tension of PFBA, PFBS, and PFOA solutions (3.33 μM each) by 24.64, 45.71, and 70.17%, respectively. PFOS exhibited little additional surface-tension response to CTAC but responded strongly to a shorter-chain QAC (C12), indicating that interfacial behavior depends on fluorocarbon–hydrocarbon chain-length compatibility. Targeted DLS, ζ-potential, and TEM characterization identified detectable bulk coassembly in selected PFAS–QAC systems at concentrations of 3.33–33.30 μM. Electrolyte addition produced PFAS-dependent changes in interfacial behavior, while NOM reduced the interfacial activity of several mixtures. These findings provide mechanistic insight into PFAS–QAC behavior relevant to the development of QAC-assisted PFAS separation processes.

## Graphical abstract

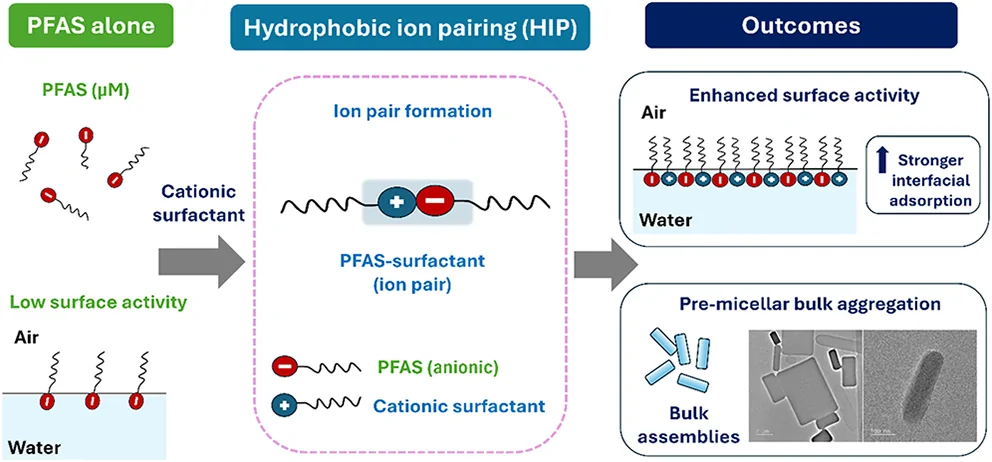

## 1 Introduction

Per- and polyfluoroalkyl substances (PFAS) are persistent environmental contaminants detected ubiquitously across environmental compartments. A 2023 U.S. Geological Survey (USGS) study estimated that at least one PFAS could be detected in about 45% of U.S. tap water samples.^1^ Due to concerns about human health effects, the U.S. Environmental Protection Agency (EPA) has established legally enforceable Maximum Contaminant Levels (MCLs) for some PFAS in drinking water at low parts-per-trillion levels,^2^ and has designated perfluorooctanoic acid (PFOA) and perfluorooctanesulfonic acid (PFOS), along with their salts and certain isomers, as hazardous substances under the Comprehensive Environmental Response, Compensation, and Liability Act (CERCLA).^3^ These regulatory actions require water providers to remove these persistent chemicals from drinking water and necessitate remediation and cleanup of PFAS-contaminated sites.

Granular activated carbon (GAC), ion-exchange (IX), and reverse osmosis (RO) have been shown to achieve high removal efficiencies for long-chain PFAS.^4^ However, GAC removes short-chain PFAS such as perfluorobutanoic acid (PFBA) and perfluorobutanesulfonic acid (PFBS) much less effectively than long-chain analogues, with early breakthrough and short bed-life reported across multiple studies.^5,6^ Foam fractionation similarly underperforms for short-chain variants.^7^ This limited performance for short-chain PFAS across treatment processes is attributed to their higher solubility, lower hydrophobicity, and lower surface activity compared to longer-chain counterparts.^8^

A growing strategy to address this gap is the addition of quaternary ammonium compounds (QACs), such as cetyltrimethylammonium chloride (CTAC) and cetyltrimethylammonium bromide (CTAB). Adding these surfactants has been shown to improve short-chain PFAS removal during coagulation,^9^ GAC filtration,^10,11^ and foam fractionation.^8,12^ For instance, with CTAC/CTAB, the removal of PFBA and PFBS increased up to >80% in foam fractionation systems,^8,12^ while CTAC-modified GAC adsorption achieved >50% removal of PFBA and >90% removal of PFBS.^11^

The enhanced removal of short-chain PFAS is attributed to the formation of ion-pair complexes between the negatively charged PFAS and positively charged surfactants. These complexes exhibit greater surface activity and hydrophobicity than the individual components.^11^ This association, commonly referred to as hydrophobic ion pairing (HIP), promotes stronger interactions between PFAS–QAC complexes and activated carbon surfaces, flocs, and air–water interfaces, thereby improving removal in GAC filtration, coagulation, and foam fractionation. However, two fundamental questions remain unresolved. It is unclear whether QACs simply modify surface charge at existing surfaces or whether HIP produces bulk supramolecular aggregates that fundamentally alter PFAS phase behavior, and whether these processes occur at concentrations relevant to real treatment and environmental systems.

Anionic-cationic (catanionic) surfactant mixtures are well-known to form thermodynamically stable supramolecular structures such as bilayer, lamellae, vesicles, micelles, and other ordered mesophases, often with dramatically reduced critical micelle concentrations (CMCs) and enhanced surface activity.^13–19^ However, much of the prior work has examined surfactant concentrations in the millimolar range, where extensive ion pairing and aggregate formation are thermodynamically favorable. In contrast, PFAS in water systems typically occur at nanomolar to micromolar levels. Although recent studies have demonstrated that adding cationic QACs can significantly enhance PFAS removal, far less is known about whether PFAS–QAC pairing and bulk aggregation occur under treatment-relevant conditions, especially for short-chain PFAS. In addition, most prior catanionic studies have been performed in relatively simple electrolyte solutions, whereas real waters contain varying pH, ionic strength, and natural organic matter (NOM), which can influence PFAS–QAC electrostatic interactions and ion pairing.

Against this background, how PFAS and QAC molecular structures jointly influence interfacial and colloidal behavior under low-micromolar, sub-CMC conditions remains poorly understood. Here, we systematically evaluate interfacial behavior across selected PFAS differing in chain length and headgroup, a homologous series of QAC alkyl-chain lengths, PFAS:QAC molar ratios, and solution chemistries under sub-CMC conditions. We complement these measurements with targeted DLS, ζ-potential, and TEM characterization to examine associated changes in colloidal behavior. By integrating surface-tension measurements with targeted colloidal characterization, we identify structure-dependent interfacial responses and detectable bulk coassembly in selected PFAS–QAC systems. These findings provide mechanistic insight into PFAS–QAC behavior relevant to the development of QAC-assisted PFAS separation processes.

## 2 Experimental Section

### 2.1 Reagents and Chemicals

Neat PFBA (CAS#375–22–4), PFBS (CAS#375–73–5), and PFOA (CAS#335–67–1) standards were purchased from Sigma-Aldrich, while PFOS (potassium salt) (CAS#2795–39–3) was purchased from Synquest Laboratories. QACs included butyltrimethylammonium chloride (BTAC, C4; Combi-Blocks Inc.; CAS#14251–72–0), octyltrimethylammonium chloride (OTAC, C8; Sigma-Aldrich; CAS#10108–86–8), dodecyltrimethylammonium chloride (DTAC, C12; Sigma-Aldrich; CAS#112–00–5), trimethyltetradecylammonium chloride (TTAC, C14; Sigma-Aldrich; CAS#4574–04–3), 1-hexadecyltrimethylammonium chloride (CTAC, C16; BeanTown Chemical, Inc.; CAS#112–02–7), and trimethylstearylammonium chloride (STAC, C18; TCI America; CAS#112–03–8). Sodium dodecyl sulfate (SDS; CAS#151–21–3) and poly­(ethylene glycol) dodecyl ether (Brij L4; CAS#9002–92–0) were purchased from Sigma-Aldrich. Sodium hydroxide (NaOH; CAS#1310–73–2), sodium chloride (NaCl; CAS#7647–14–5), and calcium chloride (CaCl_2_; CAS#10043–52–4) were purchased from Sigma-Aldrich, and hydrochloric acid (HCl; CAS#7647–01–0) was purchased from Avantor Performance Materials, LLC. Suwannee River NOM II (reverse osmosis isolate; 2R101N) was purchased from International Humic Substances Society (IHSS). Ultrapure water (UPW; resistivity ≥18.2 MΩ·cm; Milli-Q Reference Millipore-Sigma) was used throughout. All chemicals and solutions were handled using appropriate personal protective equipment and disposed of as hazardous chemical waste in accordance with institutional guidelines.

### 2.2 PFAS–QAC Mixture Preparation

PFAS–QAC mixtures were prepared in 50 mL polypropylene centrifuge tubes. Each sample was prepared by spiking 25 mL UPW with PFAS to a final concentration of 3.33 μM, followed by QAC addition at the target molar ratio and 30 s of vortex mixing. The solution was transferred to a Petri dish and allowed to equilibrate for approximately 5 min prior to measurement (Figure S1). For CTAC ratio experiments, PFAS:CTAC molar ratios of 1:1, 1:2, 1:3, 1:5, and 1:10 were tested. For PFOS, higher CTAC excess ratios (1:20, 1:30, 1:50, and 1:100) were additionally examined.

### 2.3 Surface Tension Measurements

Surface tension was measured using a Wilhelmy-plate tensiometer (FANGRUI BZY-201). The platinum plate was rinsed with methanol (Fisher Scientific; CAS#67–56–1) and UPW, then flame-cleaned before each use to remove any residual contaminants. The instrument was calibrated prior to use using a standard calibration weight. Ten measurements were recorded per sample to obtain an average surface tension value, following the 5 min equilibration period. All measurements were conducted at room temperature (23.3 ± 0.6 °C).

### 2.4 Mechanistic Controls

To confirm that enhanced PFAS surface activity is specific to electrostatic HIP, control experiments were conducted using an anionic surfactant (sodium dodecyl sulfate, SDS) and a nonionic surfactant (polyethylene glycol dodecyl ether, Brij L4).

### 2.5 Impacts of Solution Chemistry (pH, Ionic Strength, NOM)

pH was adjusted to 3, 7, and 10 using NaOH and HCl and measured by a benchtop pH meter (MU 6100 L; VWR). Salt effects were examined using NaCl and CaCl_2_ at 0.01, 0.1, and 1 M to represent monovalent and divalent electrolyte conditions, respectively. The effect of NOM was evaluated at concentrations of 0.5, 5, and 10 mg/L.

### 2.6 Particle Size, ζ-Potential, and TEM

ζ-potential and hydrodynamic size were measured using a Litesizer DLS 500 (Anton Paar) in electrophoretic and dynamic light scattering modes. TEM grids (carbon Formvar-coated copper) were prepared by adding 5 μL sample droplets, followed by heat-drying prior to imaging on a JEM-F200 (JEOL Ltd.).

### 2.7 Real Surface Water Experiments

To evaluate PFAS–CTAC interactions under realistic water quality conditions, surface water was collected from the inlet of a drinking water treatment plant in northern New Jersey. Samples were transported on ice and stored at 4 °C prior to analysis. Key physicochemical parameters of surface water are reported in Table S1.

## 3 Results and Discussion

### 3.1 HIP is Specific to Electrostatic Cationic–Anionic Interactions

CTAC produced by far the largest reduction in surface tension for PFBA, PFBS, and PFOA among the three surfactant types tested, while SDS caused no measurable change for any PFAS (Figure S2), consistent with electrostatic repulsion preventing ion pairing between like-charged SDS and PFAS.^20^ The nonionic surfactant (Brij L4), relative to Brij-aloneat the same concentration, produced no additional reduction for short-chain PFAS and only marginal decreases for PFOA and PFOS, indicating that hydrophobic interactions are insufficient to account for the strong synergism observed in PFAS–CTAC mixtures at these low concentrations. Importantly, all PFAS-only and CTAC-only solutions produced negligible surface tension reduction relative to UPW (Table S2), confirming that the enhanced surface activity observed in PFAS–CTAC mixtures is not a generic mixed-surfactant effect but arises specifically from HIP-driven interfacial adsorption.

### 3.2 Interfacial Behavior of PFAS–QAC Mixtures

#### 3.2.1 Effect of PFAS:CTAC Molar Ratio

Relative to individual PFAS and CTAC solutions, the PFAS–CTAC mixtures showed significant surface tension reductions for PFBA, PFBS, and PFOA (Figure 1A), consistent with well-known synergism in oppositely charged catanionic systems.^21–24^ The magnitude of reduction followed the chain-length trend (PFOA > PFBS > PFBA), reflecting fluorocarbon chain hydrophobicity and stronger air–water interface partitioning.^25^

**Figure 1:**
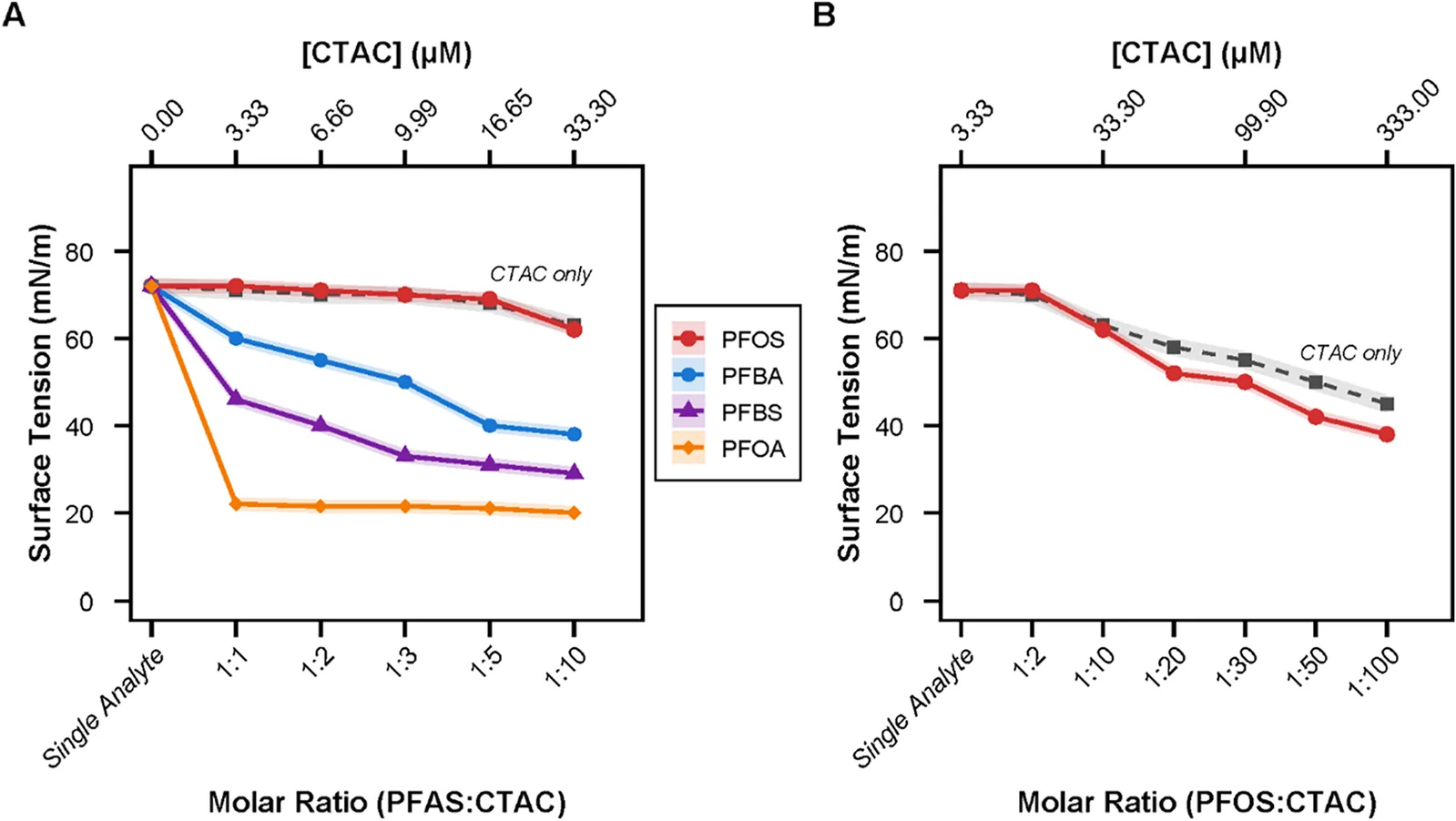
Surface tension of PFAS–CTAC mixtures as a function of PFAS:CTAC molar ratio (A) and PFOS–CTAC mixtures at higher CTAC-excessratios (B). In both panels, the secondary (top) *x*-axis indicates the absolute CTAC concentrations (μM) corresponding to each ratio. Initial PFAS and CTAC concentrations were 3.33 μM each. Shaded bands represent standard deviation (*n* = 10).

For short-chain PFAS, surface tension decreased monotonically with increasing CTAC concentration, indicating progressive formation of surface-active species (ion pairs and/or mixed aggregates). For PFOA, a minimum occurred at a 1:1 molar ratio, after which the surface tension plateaued. This is consistent with the system approaching an interfacial surface-tension minimum near charge stoichiometry, although surface-tension measurements alone cannot establish complete interfacial saturation or determine the fate of additional CTAC.

Interestingly, PFOS showed little additional surface-tension response to CTAC across the 1:1 to 1:10 PFOS:CTAC range, and the PFOS–CTAC profile was broadly similar to that of CTAC alone. DLS and ζ-potential measurements revealed a distinct intensity peak at ∼550 nm and a positive ζ-potential of +15.1 ± 4.2 mV for equimolar PFOS–CTAC at 3.33 μM, while all other PFAS–CTAC pairs at the same concentration showedno detectable colloidal signal under the measurement conditions (Table S3). PFOS alone also exhibited concentration-dependent self-aggregation (Figure S4), consistent with earlier work that reported nonmicellar bulk aggregates of PFOS at sub-CMC concentrations (>100 μM).^26^ As catanionic systems are known to further lower effective aggregation thresholds,^27^ these observations provide evidence of detectable PFOS–CTAC bulk coassembly at substantially lower concentrations. It must be noted that these techniques characterize bulk-phase aggregates and do not directly probe the interface. Therefore, the present data cannot quantify the relative bulk and interfacial populations or establish that PFOS–CTAC-associated species reside predominantly in either phase.

However, at higher CTAC excess (>1:10), PFOS–CTAC surface tension dropped below that of the CTAC-only solution (Figure 1B), indicating the emergence of additional surface-active species due to excess CTAC resolubilizing bulk structures into smaller, more mobile mixed species that can populate the interface. Similar transitions at large cationic excess are well documented in other catanionic systems.^16,27^

#### 3.2.2 Effect of QAC Chain Length

To determine whether hydrophobic tail length systematically affects PFAS–QAC HIP under micromolar, premicellar conditions, we evaluated six QACs of varying chain length (C4, C8, C12, C14, C16, and C18) at a 1:2 molar ratio (3.33 μM PFAS: 6.66 μM QAC). This ratio was selected because QACs are typically added in excess in HIP-assisted treatment studies to favor pairing and minimize unpaired PFAS.^9,11^

The chain-length dependence showed clear PFAS-specific behavior (Figure 2). For PFBA, surface tension decreased only when paired with long-chain QACs (C16, C18), consistent with PFBA’s low intrinsic hydrophobicity requiring a long hydrocarbon tail to form a sufficiently surface-active ion pair. For PFBS, reduction began at C12, reflecting its greater hydrophobicity relative to PFBA.

**Figure 2:**
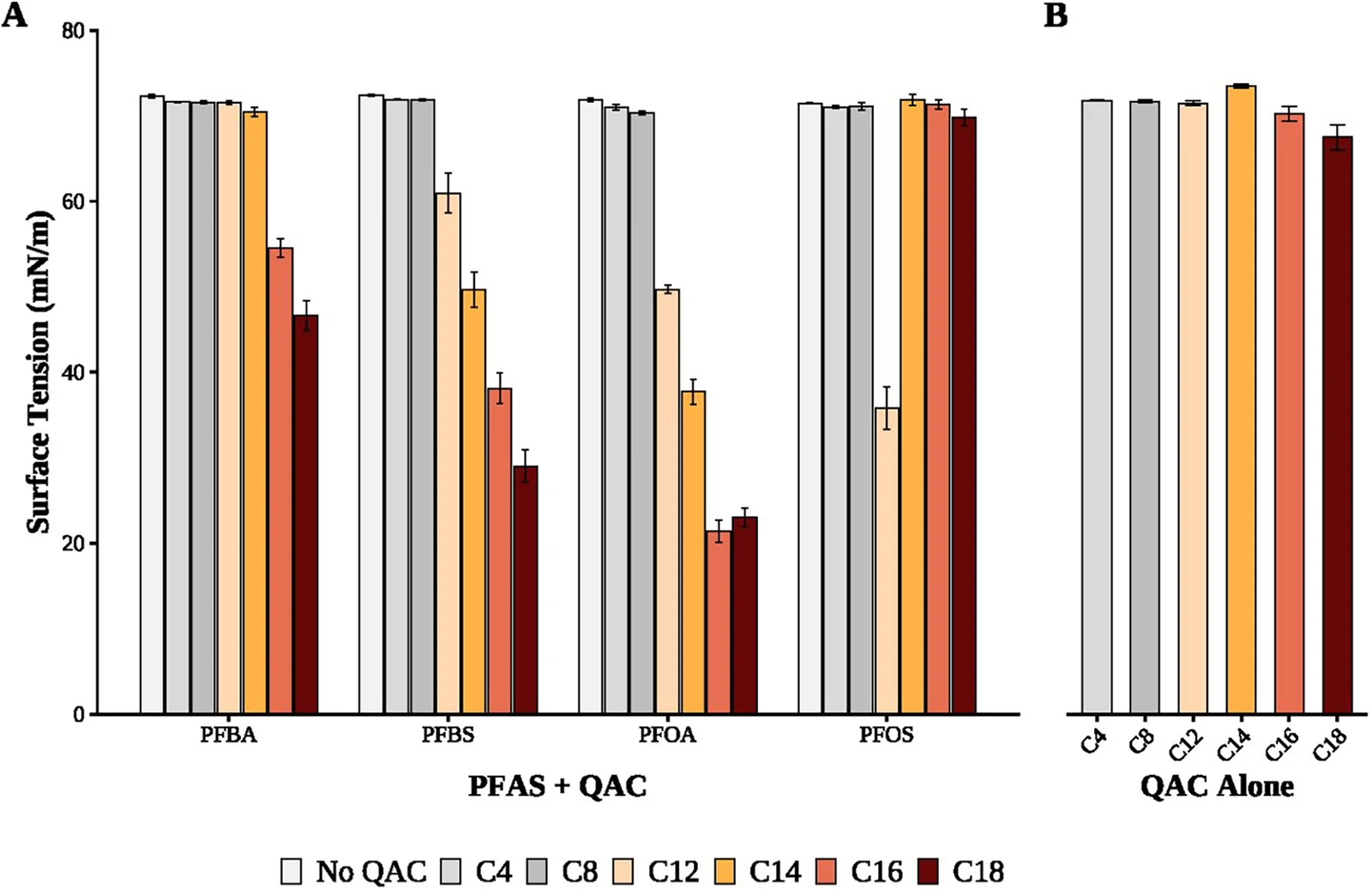
Effect of QAC alkyl chain length on surface tension of PFAS–QAC mixtures. (A) Surface tension of PFAS (3.33 μM each) in the presence of QACs with alkyl chain lengths ranging from C4 to C18 (6.66 μM each). (B) Surface tension of QAC solutions alone at 6.66 μM. Error bars represent standard deviation (*n* = 10).

For PFOA, surface tension decreased progressively from C12 through C16, with a slight increase at C18, suggesting an optimal tail length beyond which either bulk self-assembly sequesters complexes from the interface or packing frustration reduces interfacial efficiency. Similar changes in adsorption morphologies such as bulk aggregation and increased packing frustration have been observed in other catanionic systems due to hydrophobic tail-length mismatches.^27–29^

For PFOS, the C12 QAC (DTAC) was uniquely effective. Only DTAC produced an additional decrease in surface tension beyond PFOS alone, while shorter (C4, C8) and longer (C14, C16, C18) QACs did not. To determine whether these similarly weak surface-tension responses shared a common origin, we performed additional DLS and ζ-potential measurements for PFOS paired with the six QACs (Table S4), which revealed distinct structure-dependent responses. For the longer-chain QACs (C14, C16, and C18), the PFOS–QAC mixtures produced distinct particle size peaks in DLS and positive ζ-potential, indicating detectable bulk aggregation under the tested conditions. In contrast, the shorter-chain QACs (C4, C8) exhibited no detectable colloidal signal under identical concentrations. Here, the weak response likely reflects insufficient hydrophobicity to form surface-active PFOS–QAC ion pairs rather than bulk aggregation. While C12 (DTAC) also showed no observable colloidal signal changes, the decrease in surface tension at C12 indicates that PFOS–DTAC species remain sufficiently soluble and mobile to populate the interface. C12 thus occupies a narrow window that avoids bulk sequestration while still pairing effectively with PFOS and may represent a chain-length “sweet spot” beyond which detectable bulk aggregation becomes more prominent as QAC chain length increases.

These results indicate that PFAS–QAC pairing and interfacial expression at low-micromolar concentrations strongly depends on hydrophobic tail matching, with short-chain PFAS requiring long-chain QACs to form more surface-active species, and long-chain PFAS exhibiting an optimum determined by the balance between ion-pair formation and bulk coassembly.

#### 3.2.3 Impact of Solution Chemistry

##### 3.2.3.1 pH

pH had a minimal effect on surface tension for all PFAS–CTAC mixtures across pH 3–10 (Figure 3A), indicating that PFAS–CTAC association and surface activity are largely pH-independent under the conditions tested. This weak pH dependence is expected because CTAC carries a permanently charged quaternary ammonium headgroup,^30^ and recent NMR-based acid dissociation constant (p*K*_a_) measurements indicate that PFBS, PFOA, and PFOS are essentially fully deprotonated across this pH range.^31^ Thus, the electrostatic driving force for HIP does not change meaningfully with pH under the conditions studied. This suggests PFAS–CTAC interactions remain robust across environmentally relevant pH conditions at low micromolar concentrations.

**Figure 3:**
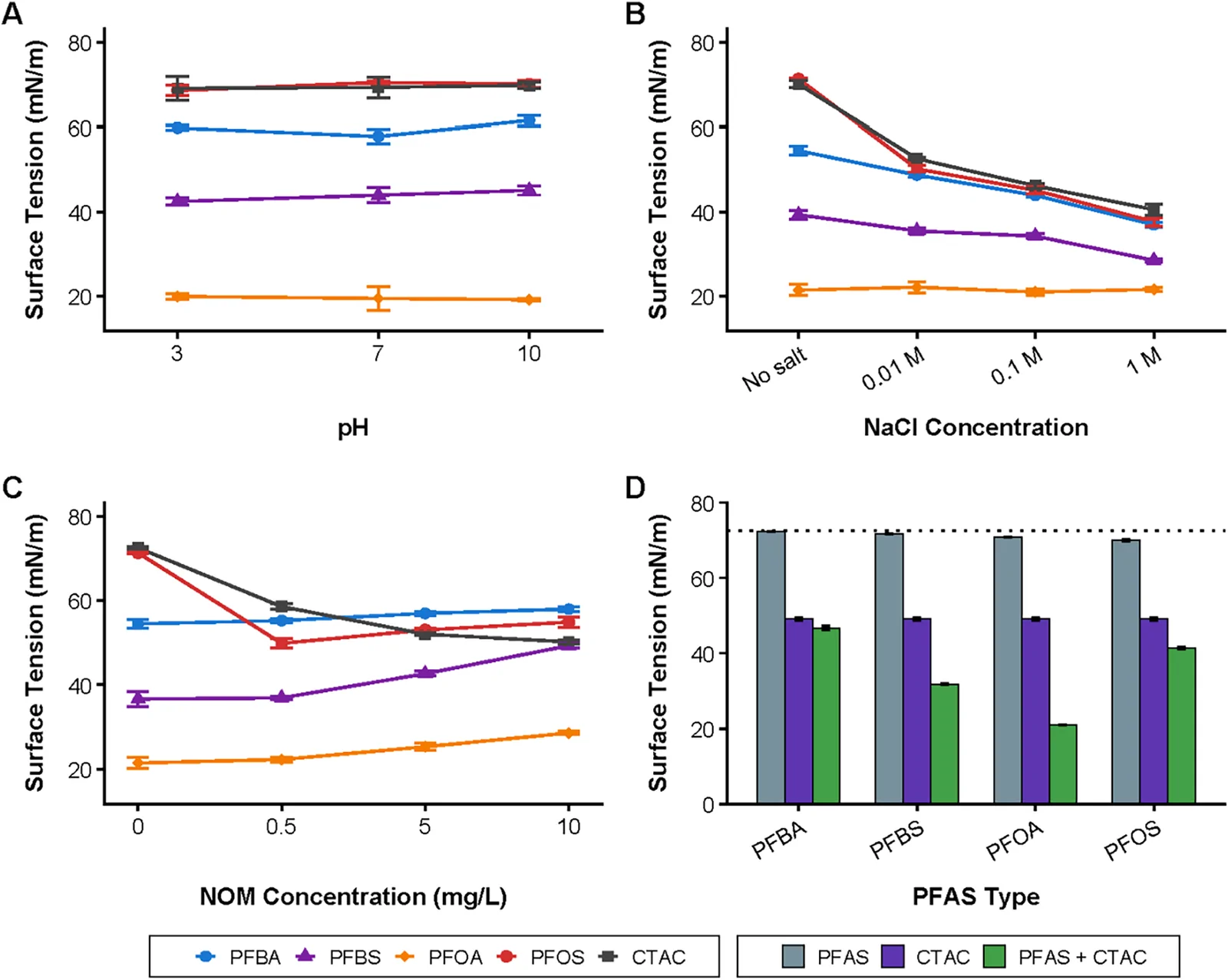
Surface tension of PFAS–CTAC mixtures as a function of (A) pH, (B) NaCl concentration, and (C) NOM concentration, and (D) PFAS- and CTAC-spiked real surface water, where the dotted horizontal line denotes the surface tension of untreated surface water. Concentrations were fixed at 3.33 μM PFAS and 6.66 μM CTAC (1:2 molar ratio). Error bars represent standard deviation (*n* = 10).

##### 3.2.3.2 Ionic Strength and Electrolyte Type

While added electrolyte is well-known to lower the CMC of many ionic surfactants by screening headgroup repulsions,^32^ classical electrolyte-screening^33^ and ion-pairing frameworks^34^ suggest that it may also screen the attractive interaction between oppositely charged PFAS and CTAC headgroups, potentially weakening PFAS–CTAC association and reducing its interfacial expression. We therefore examined the net effect of increasing ionic strength on the interfacial behavior of PFAS–CTAC mixtures. Increasing NaCl concentration progressively lowered the surface tension of both PFBA–CTAC and PFBS–CTAC relative to their respective no-salt conditions (Figures 3B and S3B), demonstrating that NaCl addition further increased the apparent surface activity of both mixtures. At each added NaCl concentration, both mixtures also showed lower surface tension than the concentration-matched CTAC-only solution (Figure 3B), with the difference being most pronounced for PFBS–CTAC. Baseline-referenced reductions for PFBA–CTAC and PFBS–CTAC were smaller than those for CTAC alone (Table S5). However, because CTAC alone and the PFAS–CTAC mixtures began at substantially different surface tensions, and because surface tension is not linearly related to interfacial adsorption, the ΔST values should be interpreted alongside the absolute surface tensions rather than as a direct measure of PFAS–CTAC association. Together, these results demonstrate that NaCl addition modifies PFAS–CTAC interfacial behavior, while the lower surface tensions relative to CTAC alone are consistent with a persistent PFAS-dependent contribution.

PFOA–CTAC exhibited a contrasting response, with surface tension remaining comparatively unchanged across the tested NaCl concentrations (Figure 3B). Because PFOA–CTAC already produced the lowest surface tension under the no-salt condition and showed little further reduction at PFAS:CTAC ratios above 1:1 (Figure 1A), its limited electrolyte response is consistent with the system approaching an interfacial surface-tension minimum under the tested conditions. However, surface-tension measurements alone cannot establish complete interfacial saturation or optimal molecular packing.

PFOS–CTAC showed a salt-dependent surface-tension profile broadly similar to that of CTAC alone (Figure 3B and Table S5). The close agreement between the two profiles, together with the 2-fold molar excess of CTAC, indicates that the measured electrolyte response was largely governed by CTAC, with no clear additional PFOS-dependent contribution to interfacial activity. This interpretation is consistent with the limited additional PFOS-dependent response observed in Figure 2A,B.

To assess the influence of electrolyte valence on PFAS–CTAC interfacial behavior, NaCl and CaCl_2_ were compared at matched ionic strength (Figure S3A) and matched molar concentration (Figure S3B). At equal ionic strength, NaCl produced numerically lower surface tension values than CaCl_2_ for PFBA–CTAC, PFBS–CTAC, and PFOS–CTAC mixtures, whereas no consistent difference was observed for PFOA–CTAC. At equal molar concentration, the differences varied in both magnitude and direction across PFAS and salt concentrations. These numerical differences were not sufficiently consistent to establish a systematic effect of cation identity or valence or to support a mechanistic interpretation.

##### 3.2.3.3 Natural Organic Matter (NOM)

The addition of NOM led to a monotonic increase in surface tension of PFBA–CTAC, PFBS–CTAC, and PFOA–CTAC mixtures (Figure 3C), indicating progressive loss of interfacially available PFAS–CTAC ion pairs. NOM is a heterogeneous mixture rich in negatively charged functional groups that associates strongly with cationic surfactants,^35–37^ redistributing CTAC into NOM-associated assemblies and away from PFAS. The result is fewer surface-active PFAS–CTAC complexes available for interfacial adsorption. This trend suggests that elevated NOM concentrations can limit the interfacial expression of HIP, with potential implications for PFAS removal efficiency in NOM-rich waters.

In contrast, the PFOS–CTAC mixture displayed a nonmonotonic response to NOM addition. An initial addition of 0.5 mg/L NOM produced a sharp decrease in surface tension relative to PFOS–CTAC without NOM, followed by a steady increase at higher NOM concentrations (Figure 3C). For CTAC-only, surface tension decreased monotonically with increasing NOM, indicating the formation of surface-active CTAC–NOM assemblies.

The initial PFOS–CTAC minimum compared to CTAC-only at low NOM indicates that NOM enables enhanced interfacial availability of PFOS by facilitating its incorporation into NOM-mediated assemblies, forming interfacially active NOM–PFOS–CTAC ternary complexes. At higher NOM concentrations, PFOS and CTAC become dispersed across increasing NOM sites, reducing the abundance of surface-active ternary assemblies, leading to an increase in surface tension. This suggests that NOM plays a regulatory role in controlling the distribution and interfacial adsorption of PFOS–CTAC assemblies. However, further structural characterization would be required to confirm the nature of these NOM-mediated associations.

Collectively, Sections 3.1 and 3.2 demonstrate that PFAS–QAC interfacial behavior remains strongly structure-dependent under low-micromolar, sub-CMC conditions. The observed responses cannot be explained by PFAS or QAC surface activity alone and vary with PFAS identity, QAC alkyl-chain length, and mixing ratio. These interfacial measurements also identify conditions for targeted colloidal characterization and provide the basis for evaluating whether a limited surface-tension response reflects weak association or the partitioning of PFAS–QAC-associated species into the bulk aqueous phase.

### 3.3 Colloidal Aggregation and Bulk Structure

Because surface-tension measurements characterize interfacial behavior but cannot independently determine whether PFAS–QAC-associated species remain dissolved or form bulk colloidal structures, selected mixtures were further examined using DLS, ζ-potential analysis, and TEM. DLS and ζ-potential measurements showed no detectable colloidal signal for UPW, PFAS-only, CTAC-only, PFBA–CTAC, PFBS–CTAC, and PFOA–CTAC mixtures at 3.33 μM per component (Table S3). Because DLS reports an intensity-weighted size distribution in which scattering intensity scales with the sixth power of particle diameter, smaller species (<10 nm), such as ion pairs, small clusters, or oligomeric species, contribute negligibly to the measured signal even if present at appreciable molar concentrations. Consequently, the absence of a detectable DLS peak or ζ-potential shift does not distinguish between molecularly dispersed PFAS–CTAC species and small ion pairs or transient nanoscale clusters below the instrument’s reliable detection threshold. These results therefore indicate that any PFAS–CTAC association for PFBA, PFBS, and PFOA at 3.33 μM either produces species too small or too low in number fraction to generate a measurable colloidal signature under these low-ionic-strength conditions, rather than establishing the absence of association altogether.

In contrast, the equimolar PFOS–CTAC mixture (3.33 μM each) showed a positive ζ-potential (+15.1 ± 4.2 mV) and a distinct DLS intensity peak at 560.3 ± 161.9 nm (Table S3), indicating formation of larger, positively charged aggregates at micromolar concentrations. TEM corroborated this observation, revealing a morphologically heterogeneous population of coassembled structures that were absent in PFOS- and CTAC-only controls. PFOS-only samples deposited as disordered amorphous films, whereas PFOS–CTAC samples showed discrete compact nanoparticles (∼150 nm) and larger aggregate clusters (Figure 4), consistent with the broad size distribution measured by DLS (polydispersity index (PDI): 0.35–0.52).

**Figure 4:**
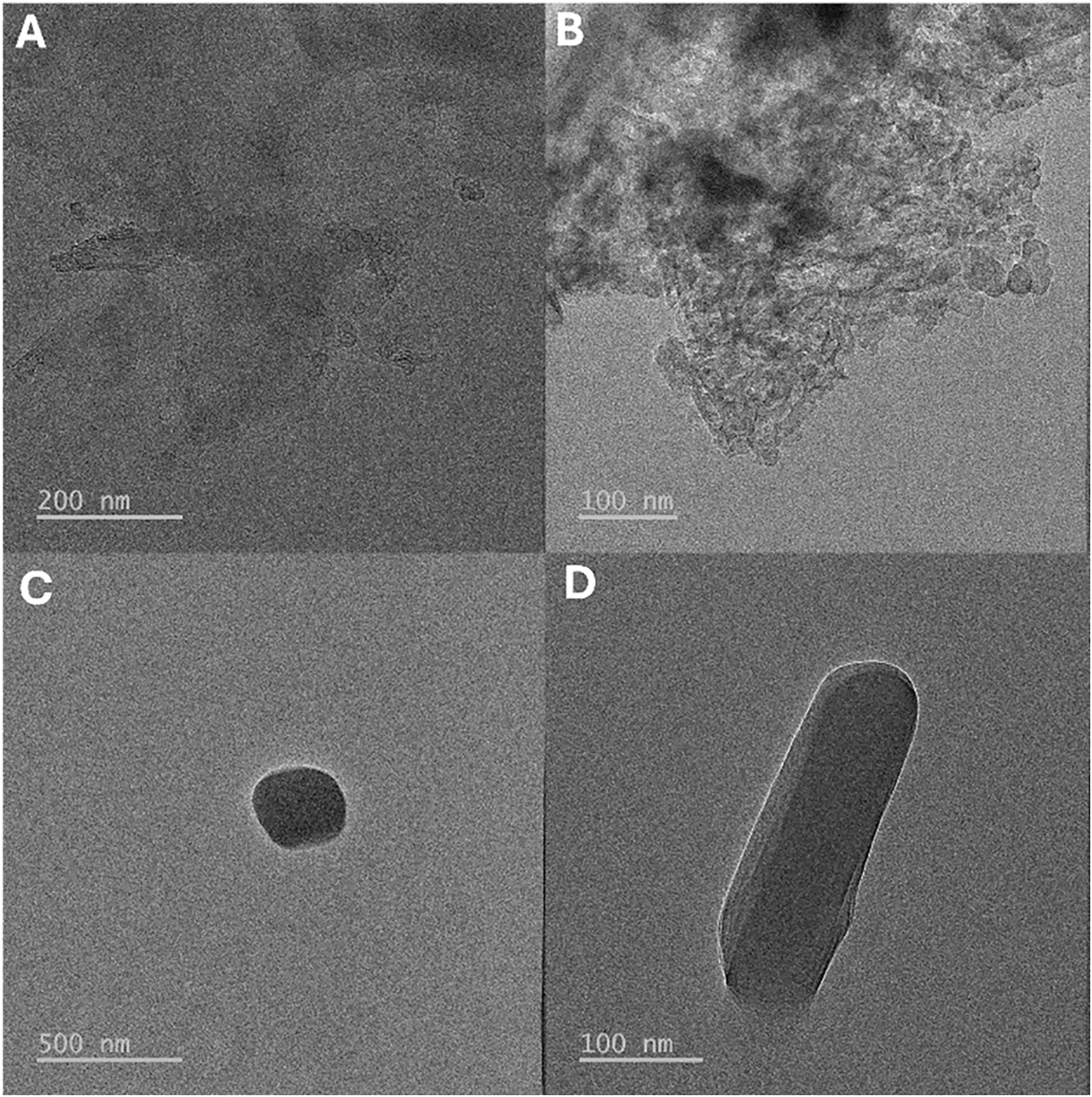
TEM characterization of PFOS–CTAC mixture at 3.33 μM each. (A) PFOS alone deposited as a disordered amorphous film with no defined morphology. (B–D) Structures observed in the PFOS–CTAC mixture and absent in PFOS- and CTAC-only solutions, including compact nanoparticles (C) and larger aggregates (B, D).

The positive ζ-potential implies a surface excess of CTA^+^ relative to PFAS at the aggregate surface, producing cation-rich mixed assemblies. This is consistent with prior catanionic studies showing nonstoichiometric internal compositions enriched in the better-packing surfactant even at stoichiometric ratios due to nonideal mixing driven by hydrophobic tail incompatibility.^16,38,39^

Recent work further indicates that PFAS alone can self-aggregate at concentrations well below historically reported CMCs and that these assemblies are not classical micelles. Sobolewski et al. reported that PFBS, PFOA, and PFOS form nonmicellar bulk aggregates at ≤1 mM (10–200 μM, depending on pH).^26^ Consistent with these thresholds, we did not observe aggregates for PFBS and PFOA at 3.33 μM, while PFOS exhibited concentration-dependent self-aggregation behavior at ≥33.30 μM (Figure S4). Because catanionic interactions further lower effective aggregation thresholds, the emergence of mixed PFOS–CTAC aggregates at low micromolar concentrations (3.33 μM) is consistent with this evolving view of PFAS self-assembly.

While Sobolewski et al. reported bulk self-aggregates of PFBS at approximately 10–100 μM, under our conditions, PFBS-only and CTAC-only solutions showed no detectable changes in ζ-potential or DLS, and no distinct structures were observed in TEM at either 3.33 or 33.30 μM. However, PFBS–CTAC mixtures at 33.30 μM exhibited a clear change in colloidal behavior. ζ-potential measurements showed a positive shift in surface charge (+30.5 ± 6.6 mV) (Figure 5A), and DLS revealed three distinct intensity-weighted peaks at ∼750, ∼2000, and ∼3000 nm (Figure 5B), suggesting a polydisperse population of coassembled structures spanning submicron to micron length scales. The broad PDI (∼0.2–0.7) is consistent with anisotropic, nonspherical geometries.^40,41^ TEM corroborated this polydispersity, revealing rectangular, plate-like structures with sharp, faceted edges of varying sizes (Figure 5C), morphologies characteristic of anisotropic, nonmicellar assemblies.^42,43^ Progressive cloudiness and eventual precipitation observed visually with increasing PFBS–CTAC concentrations from 3.33 to 3.33 mM (Figure S5) are consistent with growing aggregate size and loss of Brownian stabilization.

**Figure 5:**
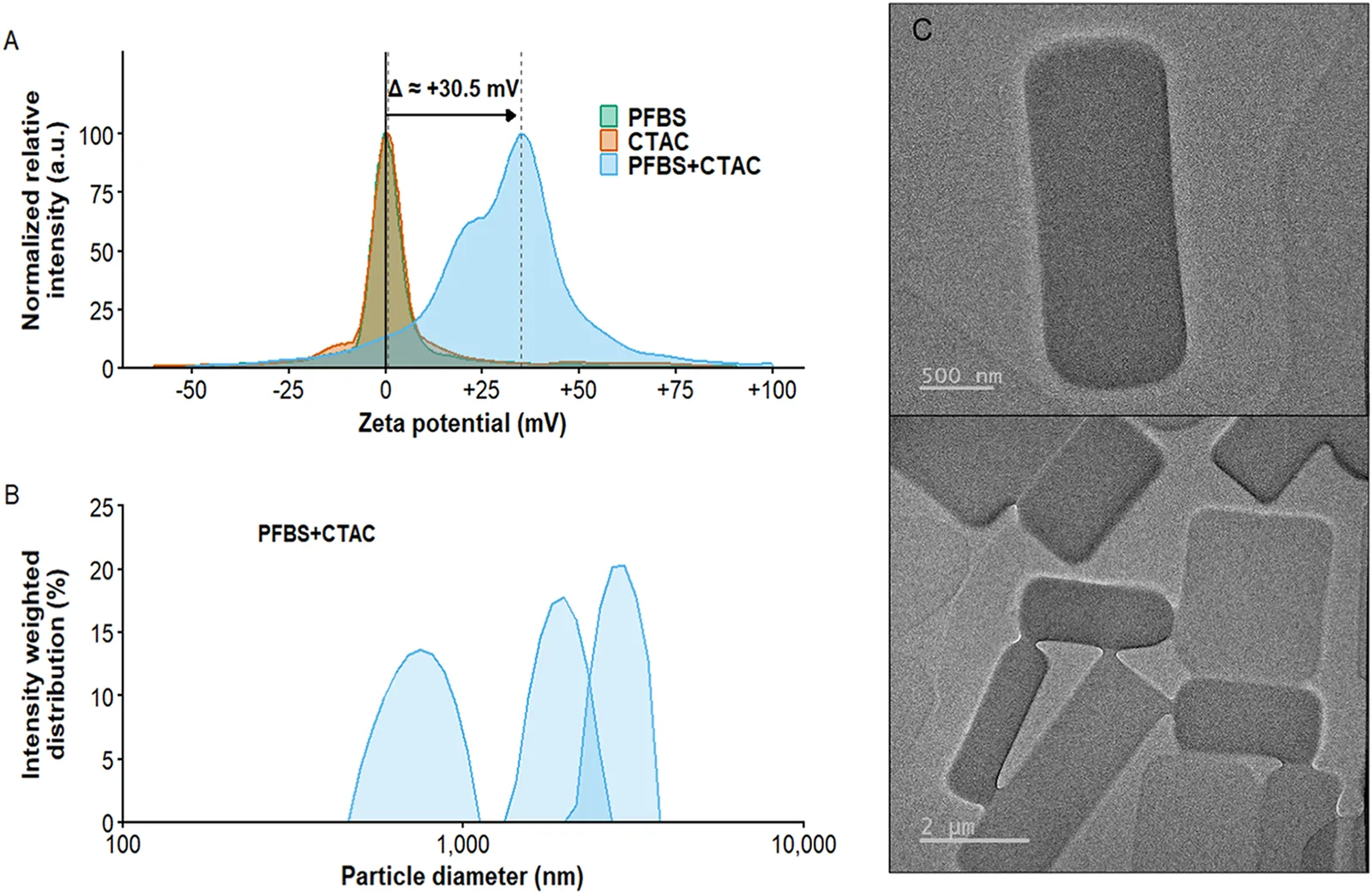
Colloidal and morphological characterization of PFBS–CTAC mixture at 33.30 μM each. (A) ζ-potential distributions of PFBS-only, CTAC-only, and PFBS–CTAC mixtures. (B) DLS intensity-weighted size distribution of the same solutions. (C) Representative TEM images of PFBS–CTAC mixture showing rectangular plate-like structures with faceted edges at two magnifications.

It must be noted that TEM grids were prepared by conventional heat-drying, which can concentrate, coalesce, or recrystallize surfactant coassemblies that are dynamic in solutions. The geometry observed here for both PFBS and PFOS may not be representative of the structures in the hydrated equilibrium state. Also, DLS reports an intensity-weighted size distribution in which even a very small number of large aggregates can dominate scattering, while nanometer-scale aggregates contribute comparatively little.^44^ DLS inherently cannot distinguish between well-defined vesicles, loose flocs, or impurities such as dust or microbubbles, and the apparent hydrodynamic diameters can be sensitive to sample handling (e.g., vortexing intensity, equilibration time). Nevertheless, the consistency between DLS, ζ-potential, and TEM-observations is suggestive of the presence of detectable bulk coassembled material, although the hydrated equilibrium morphology cannot be determined from these measurements. Previous studies have reported the formation of such morphologically distinct coassemblies in fluorocarbon-hydrocarbon catanionic mixtures at millimolar concentrations.^13,18,45–47^ Here, we provide evidence of detectable bulk coassembly in selected PFAS–CTAC systems even at low micromolar concentrations. Future structural characterization by cryo-TEM or SAXS/SANS is needed to fully resolve hydrated morphologies and internal organization of the PFAS–CTAC assemblies.

The occurrence and resulting morphologies of PFAS–CTAC assemblies are strongly dependent on chain length, headgroup chemistry, and concentrations. The absence of detectable aggregation in PFOA–CTAC relative to PFOS–CTAC at identical concentrations and pH (∼5.8), where both compounds are fully deprotonated, suggests that the sulfonate headgroup has a greater propensity for coassembly with CTAC than the carboxylate headgroup. However, chain length and headgroup effects cannot be fully distinguished without matched comparisons between PFAS differing only in headgroup type. Chain length and concentration further govern aggregate morphology, as reflected in the distinct ζ-potential values, DLS particle size distribution, and TEM-resolved structures observed for PFBS–CTAC and PFOS–CTAC mixtures. The formation of detectable PFAS–CTAC coassemblies could alter bioavailability, sediment adsorption, and interfacial partitioning. These changes may influence remediation efficacy, especially at aqueous film forming foam (AFFF)-impacted sites where elevated concentrations of both PFAS and cationic surfactants may coexist.

### 3.4 Behavior in Real Surface Water

Similar to the synthetic-water experiments, CTAC addition significantly reduced the surface tension of the real surface water tested (Figure 3D), with further reductions observed in the presence of PFAS. Surface tension followed the expected chain-length trend across PFBA–, PFBS–, and PFOA–CTAC mixtures, indicating that catanionic HIP enhanced PFAS surface activity in the tested surface water. PFOS also produced a decrease in surface tension, in contrast to its behavior in UPW where the PFOS–CTAC mixture mimicked the CTAC-only profile. This suggests that background electrolytes or NOM present in the tested surface water modulate the balance between PFOS bulk sequestration and interfacial partitioning. Overall, these results provide mechanistic support for the use of cationic surfactants in PFAS interfacial separation and are consistent with previous demonstrations of CTAC-based treatment in diverse water matrices.^8–12^

## 4 Environmental and Treatment Implications

Although our measurements were made at micromolar concentrations, HIP approach can still operate at the low PFAS levels encountered in practice because QACs are dosed in excess during treatment, where the paired fraction of PFAS is governed by the absolute concentration of available cationic surfactants. Several studies have successfully utilized HIP approach for treating contaminated water with varying PFAS levels.^8–12^ Spontaneous bulk coassembly, by contrast, is unlikely to dominate at the environmentally relevant ng/L to μg/L PFAS levels of most natural waters. This is supported by our PFBS–CTAC observations, in which coassembly and precipitation intensified with concentrations increasing from 3.33 to 3.33 mM (Figure S5), indicating that bulk association becomes progressively less favorable as concentrations fall toward environmental levels. NOM may further limit bulk coassemblies of PFAS–CTAC by competing for the available CTAC.

For engineered treatment, these coassemblies and the accompanying change in charge could influence removal through several, and at times opposing, mechanisms. The increase in hydrophobicity would generally strengthen PFAS partitioning onto sorbents and into resin matrices, as in granular activated carbon and ion exchange, whereas the effect of charge neutralization would depend on the surface charge of the medium, reducing the electrostatic attraction of PFAS to positively charged sorbents and coagulant flocs while weakening its repulsion at negatively charged membranes. Moreover, because the coassemblies observed here carry a net positive charge (from the excess cationic surfactant), they may be electrostatically attracted to, and captured by, negatively charged sorbents and membranes. The larger aggregates would favor size exclusion in membrane filtration and floc growth in coagulation, yet the same structures may block membrane pores, promote fouling, or be excluded from sorbent micropores. Foam fractionation is particularly sensitive to the balance between interfacial partitioning and bulk coassembly, as surface-active coassemblies enhance interfacial capture whereas excessive bulk sequestration, favored when the QAC chain length exceeds the optimum, may retain PFAS in solution and reduce removal. On balance, enhanced hydrophobicity appears to outweigh these opposing effects. However, the extent of PFAS–QAC association and subsequent removal is governed by chain-length matching, ionic strength, and NOM.

## 5 Conclusions

This study demonstrates that PFAS–QAC interactions produce structure-dependent changes in interfacial and colloidal behavior under low-micromolar, sub-CMC conditions. Across the selected PFAS and homologous QAC series, the magnitude of the interfacial response depended on both PFAS and QAC molecular structure. Short-chain PFAS required sufficiently hydrophobic QACs to produce substantial increases in surface activity, whereas PFOS exhibited a narrower QAC chain-length dependence. Targeted DLS, ζ-potential, and TEM characterization further revealed detectable bulk coassembly in selected PFAS–QAC systems, demonstrating that a limited surface-tension response does not necessarily indicate an absence of association. These findings show that PFAS–QAC interactions can be expressed through enhanced interfacial activity, altered colloidal behavior, or both. Solution chemistry further modulated these responses, with ionic strength producing PFAS-dependent changes in interfacial behavior and NOM reducing the interfacial activity of several mixtures. Overall, the integrated interfacial and colloidal findings provide mechanistic insight relevant to the development of QAC-assisted PFAS separation processes, while further characterization is needed to resolve the structure and behavior of these assemblies under hydrated and environmentally representative conditions.

## Supplementary Material



## Notes

Additional experimental details and methods, including surface water characterization, surface tension and salt-effect measurements, ζ-potential and hydrodynamic size data, and photographic evidence of aggregate formation (PDF)
