# Supplementary material for: Hydrophobic Ion Pairing with Cationic Surfactant Modifies Surface Activity and Colloidal Behavior of PFAS
Source: ACS ES T Water. 2026 Aug 29;6(10):6545–54. doi: 10.1021/acsestwater.6c00707 (PMC13660401; doi:10.1021/acsestwater.6c00707)
Supplement: Supplementary file 1 [file ew6c00707_si_001.pdf]

Hydrophobic Ion Pairing with Cationic Surfactant Modifies Surface Activity and Colloidal Behavior of PFAS

Bishnu Pandey, Ligaya Manalastas, Afaq Ahmad, Siwei Gu, William Pennock, Arjun K. Venkatesan *

Department of Civil & Environmental Engineering, New Jersey Institute of Technology, 323 Dr. Martin Luther King Jr. Blvd., Newark, NJ 07102, USA

* Email: arjun.venkatesan@njit.edu; Phone: (973)-596-5884

Supplementary Information

1.1 Characterization of Surface Water

Table S1: Physicochemical characterization of surface water used in experiments (mean±SD; *n=3*).

Parameter	Values
pH	7.67±0.04
Electrical conductivity	297.67±4.50 μS/cm
Temperature	21.57±0.12 ºC
UV 254 absorbance	0.12±00 cm^{-1}
Turbidity	3.35±0.11 NTU

1.2 Baseline Surface Tension Values of Different Solutions

Table S2: Baseline surface tension values for the tested solutions (mean±SD; *n=10*). PFAS and other surfactants were prepared at 3.33 μM each. Values represent the average of ten measurements.

Solution	Surface Tension (mN/m)
Ultrapure water (UPW)	72.51±0.36
Perfluorobutanoic acid (PFBA)	72.30±0.18
Perfluorobutanesulfonic acid (PFBS)	72.42±0.14
Perfluorooctanoic acid (PFOA)	71.84±0.18
Perfluorooctanesulfonic acid (PFOS)	71.45±0.07
Cetyltrimethylammonium chloride (CTAC)	70.85±1.06
Sodium dodecyl sulfate (SDS) (sodium lauryl sulfate)	72.09±0.10
Polyethylene glycol (4) dodecyl ether (Brij® L4)	47.45±0.61
Surface water	72.48±0.05

1.3 Electrokinetic and DLS Characterization of PFAS–CTAC Solutions

Table S3: Zeta potential and hydrodynamic size measurements of various solutions obtained from electrokinetic and DLS analyses. All PFAS and CTAC concentrations were 3.33 μM.

Solution type	Average zeta potential (*n=3*) (mV)	Average peak intensity (*n=3*) (nm)
UPW	ND	ND
CTAC (C16)	ND	ND
PFBA	ND	ND
PFBS	ND	ND
PFOA	ND	ND
PFOS	ND	ND
PFBA+CTAC (C16)	ND	ND
PFBS+CTAC (C16)	ND	ND
PFOA+CTAC (C16)	ND	ND
PFOS+CTAC (C16)	+15.1±4.2	560.3±161.9

Table S4: Zeta potential and hydrodynamic size measurements of PFOS–QAC solutions obtained from electrokinetic and DLS analyses. PFOS and QAC concentrations were 3.33 μM and 6.66 μM, respectively.

Solution type	Zeta potential (mV)	Peak intensity (nm)
BTAC (C4)	ND	ND
OTAC (C8)	ND	ND
DTAC (C12)	ND	ND
TTAC (C14)	ND	ND
CTAC (C16)	ND	ND
STAC (C18)	ND	ND
PFOS	ND	ND
PFOS + BTAC (C4)	ND	ND
PFOS + OTAC (C8)	ND	ND
PFOS + DTAC (C12)	ND	ND
PFOS + TTAC (C14)	+13.7±0.6	286.7±63.5
PFOS + CTAC (C16)	+21.7±1.1	520.9±90.9
PFOS + STAC (C18)	+35.0±1.6	549.2±194.1

Table S5: Absolute surface tension and change relative to the corresponding no-salt condition for CTAC-containing systems containing NaCl. $\Delta ST = ST_{(no\text{-}salt)} - ST_{(salt)}$. Positive values indicate a salt-associated reduction in surface tension. PFAS and CTAC concentrations were 3.33 μM and 6.66 μM, respectively. Values are mean ± SD of ten instrument readings.

	Surface tension (mN/m)					ΔST (mN/m)				
NaCl (M)	CTAC only	PFBA – CTAC	PFBS–CTAC	PFOA – CTAC	PFOS–CTAC	CTAC-only	PFBA–CTAC	PFBS–CTAC	PFOA–CTAC	PFOS–CTAC
0	70.12± 0.82	54.49± 1.09	39.32± 1.04	21.43± 1.32	71.31± 0.15	-	-	-	-	-
0.01	52.59± 0.33	48.75± 0.57	35.48± 0.67	22.12± 1.33	50.21± 0.77	17.53	5.74	3.84	-0.70	21.10
0.1	46.19± 0.59	43.91± 0.40	34.24± 0.59	20.99± 0.73	45.12± 0.85	23.93	10.58	5.08	0.44	26.20
1	40.53± 1.26	36.97± 0.59	28.5±0 .39	21.60± 0.51	37.65± 1.06	29.59	17.53	10.82	-0.17	33.66

1.4 Assessment of Surface-Tension Equilibration Time

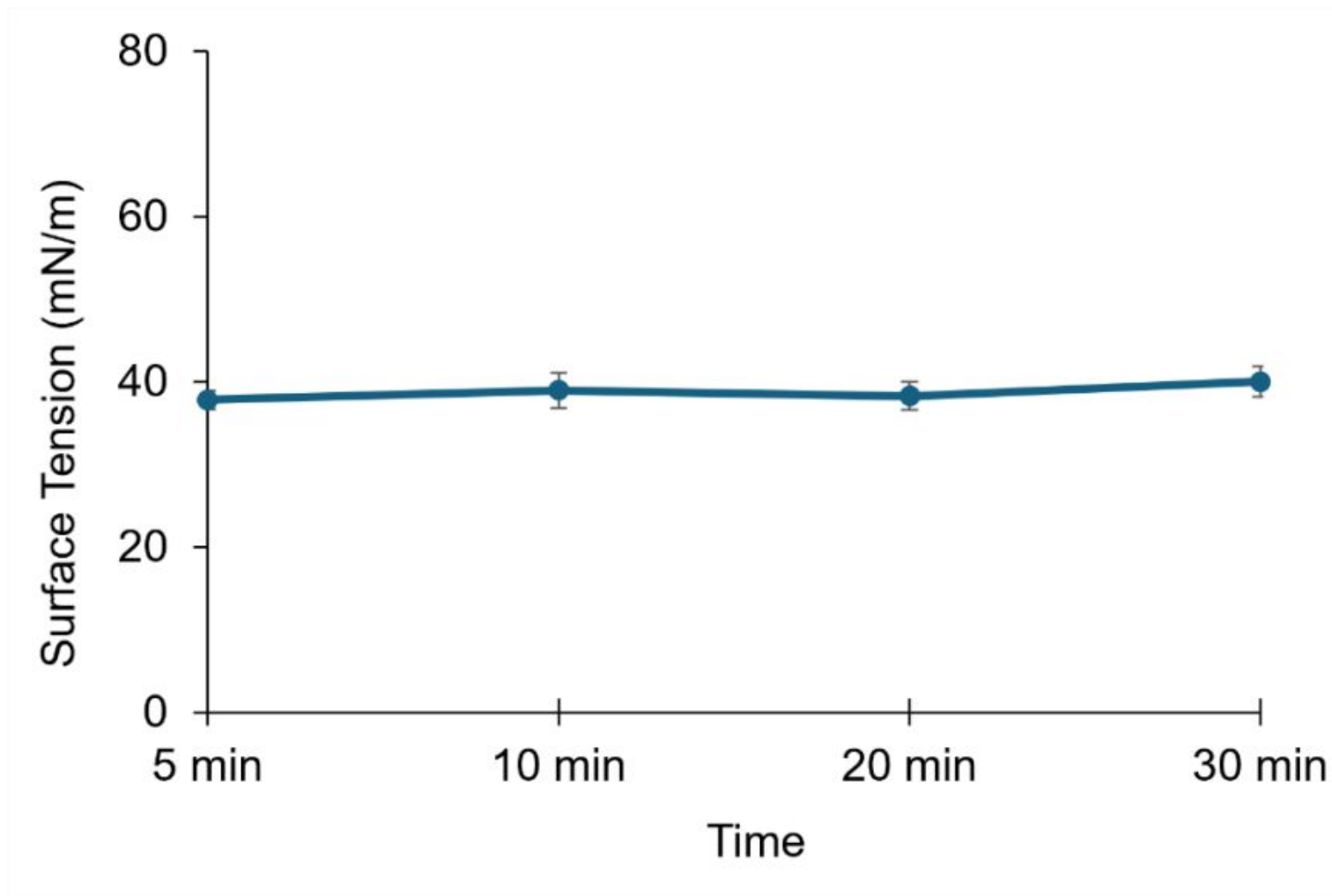

Figure S1: Time dependence of the surface tension of the PFBS–CTAC (3.33:6.66 µM) mixture. Error bars represent standard deviation (*n=10*).

1.5 Comparison with Surfactant Charge Type

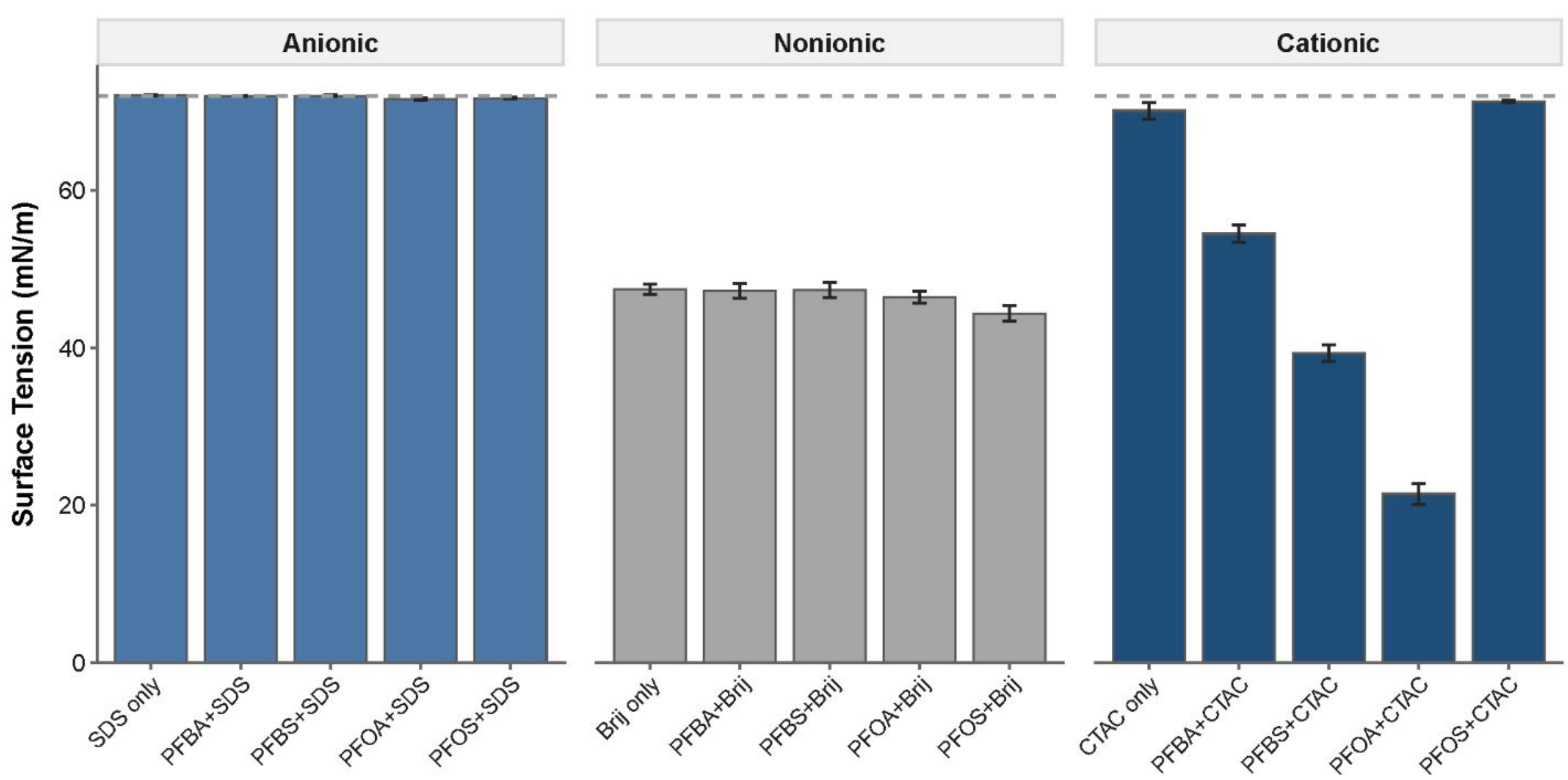

Figure S2: Surface tension of PFAS solutions in the presence of surfactants with different headgroup charges. PFAS and surfactant concentrations were 3.33 µM and 6.66 µM, respectively. CTAC, SDS, and Brij® L4 represent cationic, anionic, and nonionic surfactants. Dotted horizontal

line represents average surface tension of PFAS-only solutions. Error bars represent standard deviation (*n=10*).

1.6 Impact of Ionic Strength and Salt Valency

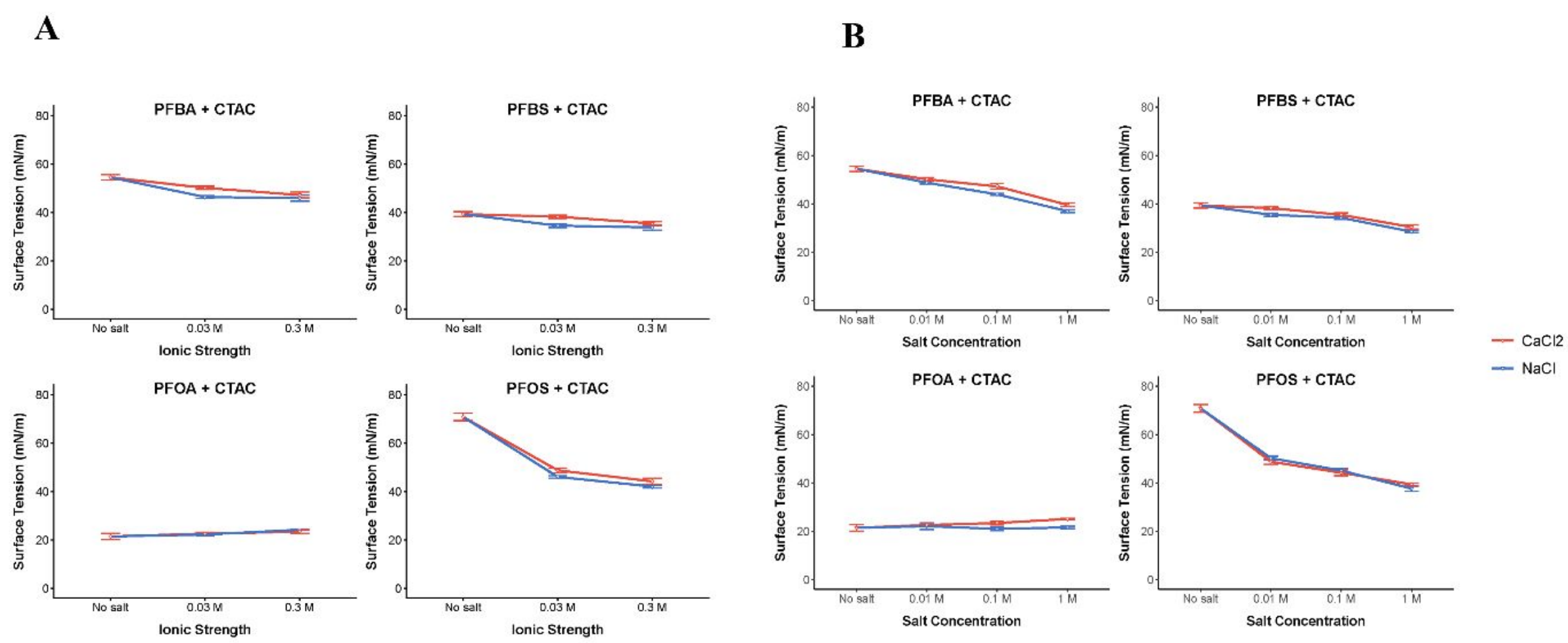

Figure S3: Surface tension of PFAS–CTAC mixtures containing 3.33 µM PFAS and 6.66 µM CTAC in the presence of NaCl and $CaCl_2$. (A) Comparison at equal ionic strength, where 0.03 and 0.3 M NaCl correspond to 0.01 and 0.1 M $CaCl_2$, respectively. (B) Comparison at equal salt molar concentration. Error bars represent standard deviation (*n=10*).

1.7 Concentration-Dependent Self-Aggregation Behavior of PFOS

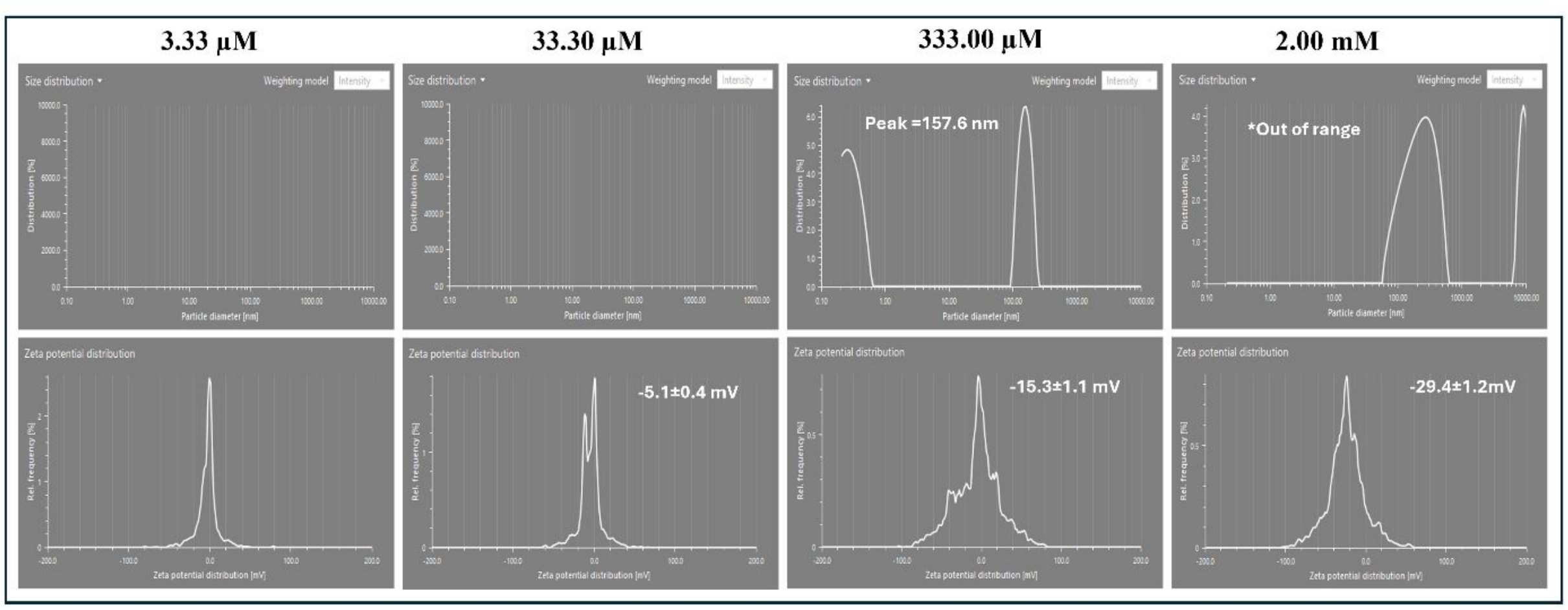

Figure S4: Hydrodynamic sizes (top row) and zeta potential (bottom row) of PFOS at varying concentrations, indicating concentration-dependent aggregation behavior.

1.8 Visual Appearance of PFBS–CTAC Mixtures

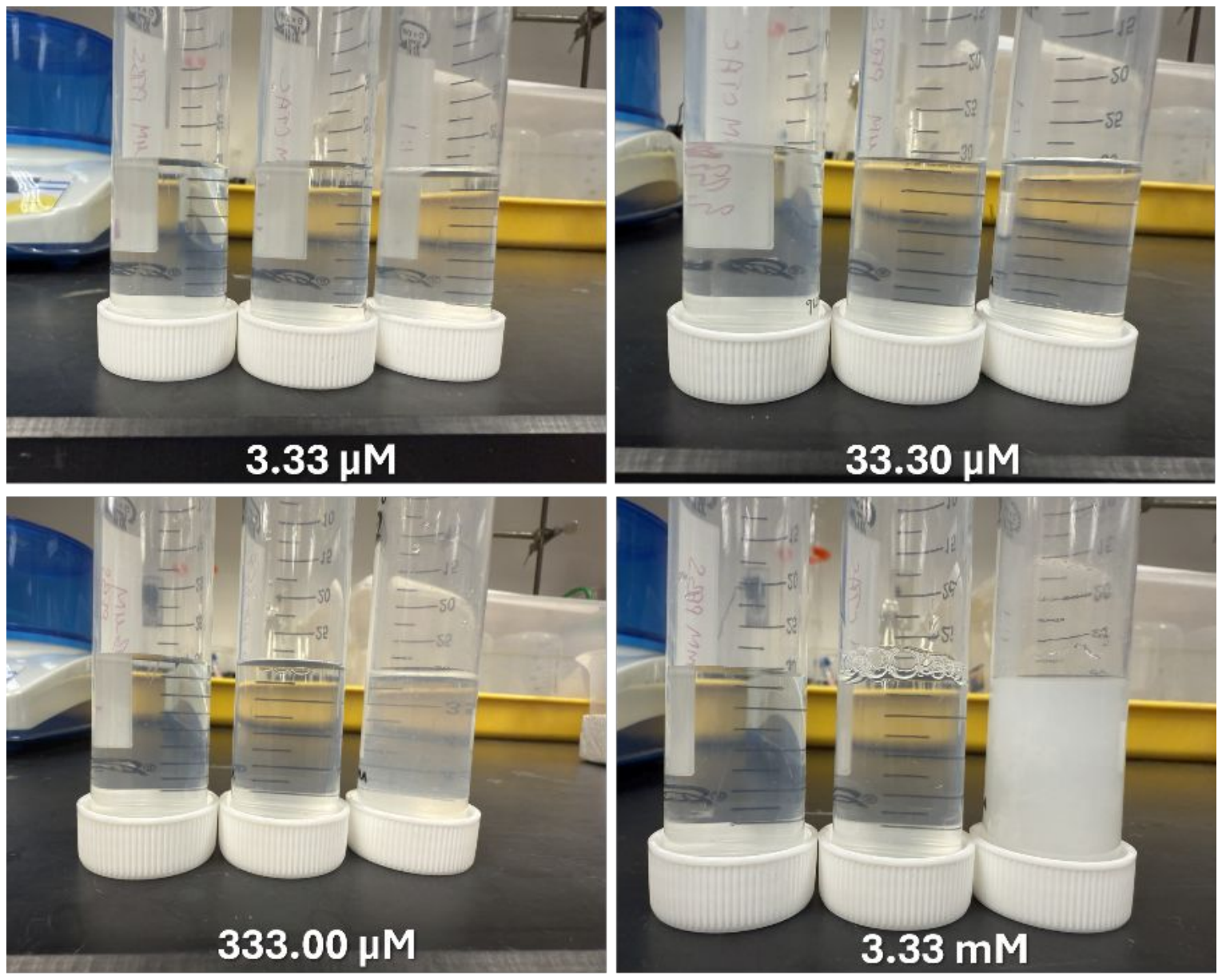

Figure S5: Visual appearance of PFBS-only, CTAC-only, and PFBS–CTAC mixtures over concentrations ranging from 3.33 μM to 3.33 mM. In each panel, vials from left to right contain PFBS, CTAC, and PFBS–CTAC mixture, respectively. Cloudiness starts to appear in the PFBS–CTAC mixtures at and above 33.30 μM, indicating the formation of bulk aggregates at higher concentrations.
